# Effectiveness of COVID-19 mRNA Vaccines Against COVID-19–Associated Hospitalization — Five Veterans Affairs Medical Centers, United States, February 1–August 6, 2021

**DOI:** 10.15585/mmwr.mm7037e3

**Published:** 2021-09-17

**Authors:** Kristina L. Bajema, Rebecca M. Dahl, Mila M. Prill, Elissa Meites, Maria C. Rodriguez-Barradas, Vincent C. Marconi, David O. Beenhouwer, Sheldon T. Brown, Mark Holodniy, Cynthia Lucero-Obusan, Gilberto Rivera-Dominguez, Rosalba Gomez Morones, Alexis Whitmire, Evan B. Goldin, Steve L. Evener, Maraia Tremarelli, Suxiang Tong, Aron J. Hall, Stephanie J. Schrag, Meredith McMorrow, Miwako Kobayashi, Jennifer R. Verani, Diya Surie, SUPERNOVA COVID-19, Surveillance Group, Surveillance Platform for Enteric and Respiratory Infectious Organisms at the VA (SUPERNOVA) COVID-19 Surveillance Group, Ghazal Ahmadi-Izadi, Joy Burnette, Rijalda Deovic, Lauren Epstein, Amy Hartley, Elena Morales, Tehquin Tanner, Nina Patel, Ashley Tunson, Katherine Elliot, Ilda Graham, Diki Lama, Ismael Pena, Adrienne Perea, Guerry Anabelle Perez, Johane Simelane, Sarah Smith, Gabriela Tallin, Amelia Tisi, Alonso Arellano Lopez, Miguel Covarrubias Gonzalez, Bashir Lengi, Dena Mansouri, Mariana Vanoye Tamez, Babak Aryanfar, Ian Lee-Chang, Chan Jeong, Anthony Matolek, Chad Mendoza, Aleksandra Poteshkina, Saadia Naeem, Madhuri Agrawal, Jessica Lopez, Theresa Peters, Geliya Kudryavtseva, Jordan Cates, Jennifer M. Folster, Anita Kambhampati, Anna Kelleher, Yan Li, Han Jia Ng, Ying Tao

**Affiliations:** ^1^CDC COVID-19 Response Team; ^2^Michael E. DeBakey, Veterans Affairs Medical Center, Houston, Texas; ^3^Department of Medicine, Baylor College of Medicine, Houston, Texas; ^4^Atlanta Veterans Affairs Medical Center, Atlanta, Georgia; ^5^Department of Medicine, Emory University School of Medicine, Atlanta, Georgia; ^6^Department of Global Health, Rollins School of Public Health, Emory University, Atlanta, Georgia; ^7^Veterans Affairs Greater Los Angeles Healthcare System, Los Angeles, California; ^8^Department of Medicine, David Geffen School of Medicine at UCLA, Los Angeles, California; ^9^James J. Peters Veterans Affairs Medical Center, Bronx, New York; ^10^Department of Medicine, Icahn School of Medicine at Mount Sinai, New York, New York; ^11^Veterans Affairs Palo Alto Health Care System, Palo Alto, California; ^12^Public Health Surveillance and Research, Department of Veterans Affairs, Washington, DC; ^13^Department of Medicine, Stanford University, Stanford, California; ^14^Karna, LLC, Atlanta, Georgia; ^15^General Dynamics Information Technology, Falls Church, Virginia.; Atlanta Veterans Affairs Medical Center, Atlanta, Georgia; Atlanta Veterans Affairs Medical Center, Atlanta, Georgia; Atlanta Veterans Affairs Medical Center, Atlanta, Georgia; Atlanta Veterans Affairs Medical Center, Atlanta, Georgia; Atlanta Veterans Affairs Medical Center, Atlanta, Georgia; Atlanta Veterans Affairs Medical Center, Atlanta, Georgia; Atlanta Veterans Affairs Medical Center, Atlanta, Georgia; Atlanta Veterans Affairs Medical Center, Atlanta, Georgia; Atlanta Veterans Affairs Medical Center, Atlanta, Georgia; James J. Peters Veterans Affairs Medical Center, Bronx, New York; James J. Peters Veterans Affairs Medical Center, Bronx, New York; James J. Peters Veterans Affairs Medical Center, Bronx, New York; James J. Peters Veterans Affairs Medical Center, Bronx, New York; James J. Peters Veterans Affairs Medical Center, Bronx, New York; James J. Peters Veterans Affairs Medical Center, Bronx, New York; James J. Peters Veterans Affairs Medical Center, Bronx, New York; James J. Peters Veterans Affairs Medical Center, Bronx, New York; James J. Peters Veterans Affairs Medical Center, Bronx, New York; James J. Peters Veterans Affairs Medical Center, Bronx, New York; Michael E. DeBakey Veterans Affairs Medical Center, Houston, Texas; Michael E. DeBakey Veterans Affairs Medical Center, Houston, Texas; Michael E. DeBakey Veterans Affairs Medical Center, Houston, Texas; Michael E. DeBakey Veterans Affairs Medical Center, Houston, Texas; Michael E. DeBakey Veterans Affairs Medical Center, Houston, Texas; Veterans Affairs Greater Los Angeles Healthcare System, Los Angeles, California; Veterans Affairs Greater Los Angeles Healthcare System, Los Angeles, California; Veterans Affairs Greater Los Angeles Healthcare System, Los Angeles, California; Veterans Affairs Greater Los Angeles Healthcare System, Los Angeles, California; Veterans Affairs Greater Los Angeles Healthcare System, Los Angeles, California; Veterans Affairs Greater Los Angeles Healthcare System, Los Angeles, California; Veterans Affairs Greater Los Angeles Healthcare System, Los Angeles, California; Veterans Affairs Palo Alto Health Care System, Palo Alto, California; Veterans Affairs Palo Alto Health Care System, Palo Alto, California; Veterans Affairs Palo Alto Health Care System, Palo Alto, California; Veterans Affairs Palo Alto Health Care System, Palo Alto, California; CDC; CDC; CDC; CDC; CDC; CDC; CDC

COVID-19 mRNA vaccines (Pfizer-BioNTech and Moderna) have been shown to be highly protective against COVID-19–associated hospitalizations (*1*–*3*). Data are limited on the level of protection against hospitalization among disproportionately affected populations in the United States, particularly during periods in which the B.1.617.2 (Delta) variant of SARS-CoV-2, the virus that causes COVID-19, predominates (*2*). U.S. veterans are older, more racially diverse, and have higher prevalences of underlying medical conditions than persons in the general U.S. population (*2*,*4*). CDC assessed the effectiveness of mRNA vaccines against COVID-19–associated hospitalization among 1,175 U.S. veterans aged ≥18 years hospitalized at five Veterans Affairs Medical Centers (VAMCs) during February 1–August 6, 2021. Among these hospitalized persons, 1,093 (93.0%) were men, the median age was 68 years, 574 (48.9%) were non-Hispanic Black (Black), 475 were non-Hispanic White (White), and 522 (44.4%) had a Charlson comorbidity index score of ≥3 (*5*). Overall adjusted vaccine effectiveness against COVID-19–associated hospitalization was 86.8% (95% confidence interval [CI] = 80.4%–91.1%) and was similar before (February 1–June 30) and during (July 1–August 6) SARS-CoV-2 Delta variant predominance (84.1% versus 89.3%, respectively). Vaccine effectiveness was 79.8% (95% CI = 67.7%–87.4%) among adults aged ≥65 years and 95.1% (95% CI = 89.1%–97.8%) among those aged 18–64 years. COVID-19 mRNA vaccines are highly effective in preventing COVID-19–associated hospitalization in this older, racially diverse population of predominately male U.S. veterans. Additional evaluations of vaccine effectiveness among various age groups are warranted. To prevent COVID-19–related hospitalizations, all eligible persons should receive COVID-19 vaccination.

During February 1–August 6, 2021, adults aged ≥18 years hospitalized at five VAMCs (in Atlanta, Georgia; Bronx, New York; Houston, Texas; Los Angeles, California; and Palo Alto, California) were screened for inclusion in this test-negative case-control assessment.^†^ Patients were eligible for inclusion if they had COVID-19–like illness (i.e., fever, new or worsened cough or shortness of breath, loss of taste or smell, oxygen saturation on room air <94%, requirement for noninvasive ventilation or endotracheal intubation with mechanical ventilation, or chest radiograph or computed tomography pulmonary findings consistent with pneumonia) (*1*) and a molecular test (reverse transcription–polymerase chain reaction [RT-PCR] or isothermal nucleic acid amplification test) for SARS-CoV-2 performed within 14 days before admission or during the first 72 hours of hospitalization. The first SARS-CoV-2 test within this eligibility period was considered the qualifying test. Patients with COVID-19–like illness who received a positive SARS-CoV-2 test result were included as case-patients, and those with COVID-19–like illness with negative SARS-CoV-2 test results were included as controls.

Electronic health records were reviewed to obtain data on demographic characteristics, underlying medical conditions, presenting illness, SARS-CoV-2 test results, COVID-19 vaccination history, and clinical course during hospitalization. In the Atlanta and Houston VAMCs, COVID-19 vaccination status was further verified through a review of state immunization registries. Full vaccination was defined as receipt of both doses of an mRNA vaccine (Pfizer-BioNTech or Moderna) ≥14 days before the qualifying SARS-CoV-2 test. Participants who received only 1 dose of an mRNA COVID-19 vaccine, 2 mRNA doses with receipt of the second dose <14 days before the qualifying SARS-CoV-2 test, mixed mRNA vaccine products (i.e., a different product for each dose), or the Janssen (Johnson & Johnson) COVID-19 vaccine were excluded from the analysis. Available residual clinical respiratory specimens were collected from case-patients at all sites and sent to CDC for testing. Specimens were tested using CDC’s 2019-Novel Coronavirus RT-PCR Diagnostic Panel^§^; those with cycle threshold values <33 were submitted for SARS-CoV-2 whole genome sequencing (*6*). In addition, results from SARS-CoV-2 whole genome sequencing conducted by VAMC laboratories on clinical specimens from Atlanta, Palo Alto, and Bronx VAMCs were also reported to CDC.

Vaccine effectiveness (1 – adjusted odds ratio [aOR] × 100)^¶^ to prevent COVID-19–associated hospitalization was estimated by using multivariable logistic regression to compare the odds of full vaccination between case-patients and controls. Models were adjusted for VAMC site, admission date and age (with the use of cubic splines), sex, and race/ethnicity. Additional factors were included if they changed the aOR by ≥5% when added individually to the base model. Vaccine effectiveness was compared between subgroups using 95% confidence intervals (CIs). Analyses were conducted using SAS (version 9.4; SAS Institute). Protocols were reviewed and approved by the VAMC Research and Development Committee at each site. The activity was also reviewed by CDC and conducted consistent with applicable federal law and CDC policy.**

During February 1–August 6, 2021, a total of 1,494 hospitalized U.S. veterans met inclusion criteria. After excluding 319 ineligible persons (67 with missing demographic data or vaccination date or product information, 230 who received only 1 dose of mRNA COVID-19 vaccine or 2 doses <14 days before the qualifying SARS-CoV-2 test, one who received mixed mRNA COVID-19 vaccine products, and 21 who received the Janssen COVID-19 vaccine), 388 case-patients and 787 controls were included in the analysis. Among these 1,175 patients, 1,093 (93.0%) were men, the median age was 68 years (interquartile range [IQR] = 59–75 years), 574 (48.9%) were Black, and 93 (7.9%) were Hispanic (Table 1). Prevalence of underlying medical conditions was high and included obesity (46.8%), diabetes (43.8%), atherosclerotic cardiovascular disease (29.2%), and chronic obstructive pulmonary disease (25.4%) (Table 1). Overall, 54 (13.9%) case-patients and 378 (48.0%) controls were fully vaccinated. Among fully vaccinated persons, the median interval between the second COVID-19 vaccine dose and the qualifying SARS-CoV-2 test was 83 days (IQR = 49–129). Among 171 case-patients with SARS-CoV-2 lineage determined,^††^ Delta became the predominant variant across all sites in July 2021 (Figure).

**TABLE 1:** Characteristics of COVID-19 case-patients and controls among hospitalized veterans — five Veterans Affairs Medical Centers, United States, February 1–August 6, 2021

Characteristic	No. (%)		
	Total (N = 1,175)	Case-patients (n = 388)	Controls (n = 787)
**Male sex**	**1,093 (93.0)**	**353 (91.0)**	**740 (94.0)**
**Age, yrs, median (IQR)**	**68 (59–75)**	64 (53–73)	69 (62–76)
**Age group, yrs**			
18–49	**132 (11.2)**	74 (19.1)	58 (7.4)
50–64	**342 (29.1)**	125 (32.2)	217 (27.6)
65–74	**401 (34.1)**	110 (28.4)	291 (37.0)
75–84	**207 (17.6)**	53 (13.7)	154 (19.6)
≥85	**93 (7.9)**	26 (6.7)	67 (8.5)
**Race/Ethnicity**			
Black, non-Hispanic	**574 (48.9)**	195 (50.3)	379 (48.2)
White, non-Hispanic	**475 (40.4)**	141 (36.3)	334 (42.4)
Hispanic, any race	**93 (7.9)**	40 (10.3)	53 (6.7)
Other, non-Hispanic*	**33 (2.8)**	12 (3.1)	21 (2.7)
**Resident in long-term care facility^†^** (unknown = 58)	**66 (5.9)**	14 (3.8)	52 (7.0)
**VAMC study site**			
Atlanta, Georgia	**362 (30.8)**	121 (31.2)	241 (30.6)
Bronx, New York	**83 (7.1)**	26 (6.7)	57 (7.2)
Houston, Texas	**410 (34.9)**	180 (46.4)	230 (29.2)
Los Angeles, California	**223 (19.0)**	44 (11.3)	179 (22.7)
Palo Alto, California	**97 (8.3)**	17 (4.4)	80 (10.2)
**Month of hospital admission**			
February	**275 (23.4)**	101 (26.0)	174 (22.1)
March	**174 (14.8)**	51 (13.1)	123 (15.6)
April	**202 (17.2)**	63 (16.2)	139 (17.7)
May	**138 (11.7)**	29 (7.5)	109 (13.9)
June	**99 (8.4)**	26 (6.7)	73 (9.3)
July	**224 (19.1)**	87 (22.4)	137 (17.4)
August	**63 (5.4)**	31 (8.0)	32 (4.1)
**Fully vaccinated for COVID-19^§^**	**432 (36.8)**	54 (13.9)	378 (48.0)
**COVID-19 vaccine product among fully vaccinated**			
BNT162b2 (Pfizer-BioNTech)	**285 (66.0)**	43 (79.6)	242 (64.0)
mRNA-1273 (Moderna)	**147 (34.0)**	11 (20.4)	136 (36.0)
**Days between second vaccine dose and SARS-CoV-2 test among fully vaccinated, median (IQR)**	**83 (49–129)**	126 (68–144)	77 (47–123)
**Underlying medical condition**			
Cardiovascular			
Atherosclerotic cardiovascular disease^¶^	**335 (29.2)**	78 (20.9)	257 (33.2)
Atrial fibrillation	**168 (14.3)**	50 (12.9)	118 (15.0)
Congestive heart failure	**289 (24.6)**	54 (13.9)	235 (29.9)
Hypertension	**822 (70.0)**	258 (66.5)	564 (71.7)
Venous thromboembolism**	**69 (5.9)**	20 (5.2)	49 (6.2)
Metabolic			
Diabetes	**515 (43.8)**	162 (41.8)	353 (44.9)
Dyslipidemia	**464 (39.5)**	152 (39.2)	312 (39.6)
Obesity^††^ (unknown = 3)	**549 (46.8)**	208 (53.9)	341 (43.4)
Pulmonary			
Asthma	**86 (7.3)**	19 (4.9)	67 (8.5)
Chronic obstructive pulmonary disease or emphysema	**299 (25.4)**	55 (14.2)	244 (31.0)
Obstructive sleep apnea	**214 (18.2)**	75 (19.3)	139 (17.7)
Neurologic			
Dementia	**79 (6.7)**	25 (6.4)	54 (6.9)
Stroke or transient ischemic attack	**125 (10.6)**	33 (8.5)	92 (11.7)
Renal			
Chronic kidney disease	**239 (20.3)**	66 (17.0)	173 (22.0)
End stage kidney disease on dialysis	**59 (5.0)**	14 (3.6)	45 (5.7)
Liver			
Liver disease	**113 (9.6)**	28 (7.2)	85 (10.8)
Immunocompromising condition			
Immunocompromising condition or therapy^§§^	**212 (18.4)**	36 (9.6)	176 (22.7)
**Charlson comorbidity index score^¶¶^**			
0	**215 (18.3)**	120 (30.9)	95 (12.1)
1–2	**438 (37.3)**	146 (37.6)	292 (37.1)
3–4	**306 (26.0)**	81 (20.9)	225 (28.6)
≥5	**216 (18.4)**	41 (10.6)	175 (22.2)
Tobacco use***			
Current	**242 (20.6)**	49 (12.6)	193 (24.5)
Former	**365 (31.1)**	93 (24.0)	272 (34.6)
**Hospitalizations in past year** (unknown = 31)			
0	**671 (58.7)**	267 (70.6)	404 (52.7)
1	**238 (20.8)**	66 (17.5)	172 (22.5)
2	**93 (8.1)**	18 (4.8)	75 (9.8)
≥3	**142 (12.4)**	27 (7.1)	115 (15.0)
**Intensive care unit admission** (unknown = 29)	**242 (21.0)**	85 (23.2)	157 (20.1)
**Death** (unknown = 28)	**61 (5.3)**	28 (7.7)	33 (4.2)

**Abbreviations:** IQR = interquartile range; VAMC = Veterans Affairs Medical Center.

* Includes non-Hispanic American Indian and Alaska Native, non-Hispanic Asian and Pacific Islander, non-Hispanic multiple-race, and non-Hispanic other race persons.

^†^ Includes residence before admission at VAMC and non-VAMC nursing facilities as well as other VAMC long-term housing (e.g., Domiciliary Care Program facilities).

^§^ COVID-19 vaccination status includes unvaccinated, defined as no receipt of any COVID-19 vaccine, and fully vaccinated, defined as receipt of both doses of an mRNA COVID-19 vaccine (Pfizer-BioNTech or Moderna) ≥14 days before the first SARS-CoV-2 test performed within 14 days before admission or during the first 72 hours of hospitalization.

**^¶^** Includes coronary artery disease, myocardial infarction, peripheral vascular disease, and carotid artery stenosis.

** Includes history of deep venous thrombosis and pulmonary embolism

^††^ Body mass index ≥30 kg/m^2^.

^§§^ Includes HIV/AIDS, malignancy, history of solid organ or stem cell transplant, ulcerative colitis, Crohn’s disease, systemic lupus erythematosus, rheumatoid arthritis, systemic sclerosis, and receipt of immunosuppressive therapy (systemic steroids, chemotherapy, or other immunosuppressive therapy) within 1 month of SARS-CoV-2 test.

^¶¶^ Modified from King JT, Jr., Yoon J, Rentsch CT, et al. Development and validation of a 30-day mortality index based on pre-existing medical administrative data from 13,323 COVID-19 patients: The Veterans Health Administration COVID-19 (VACO) Index. PLoS ONE 2020*;*15:e0241825.

*** Tobacco use was defined as smoking cigarettes, cigars, or pipes. Current tobacco use was use within the 12 months before hospitalization; former use was >12 months before hospitalization.

**FIGURE:**
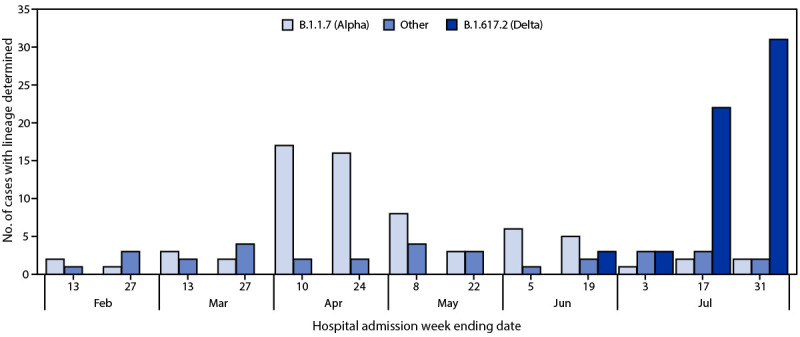
SARS-CoV-2 whole genome sequencing lineage results* for specimens from veterans aged ≥18 years hospitalized with COVID-19 — five Veterans Affairs Medical Centers,† United States, February 1–August 6, 2021^§^ * Residual clinical respiratory specimens with SARS-CoV-2 detected by reverse transcription–polymerase chain reaction with a cycle threshold <33 for at least one of two nucleocapsid gene targets were submitted for whole genome sequencing using a combination of Sanger and Illumina sequencing to maximize genome coverage. In addition, sequencing conducted at Veterans Affairs Medical Center laboratories (Clear Labs platform and Thermo Fisher Scientific Ion Torrent next-generation sequencing platform) were also included. The percentage of case-patient specimens sequenced varied over time and was lowest during February–March 2021. ^†^ Atlanta, Georgia; Bronx, New York; Houston, Texas; Los Angeles, California; and Palo Alto, California. ^§^ Sequencing conducted through July 31, 2021.

The adjusted effectiveness of full vaccination in preventing COVID-19–associated hospitalization during the entire evaluation period (February 1–August 6, 2021) was 86.8% (95% CI = 80.4%–91.1%) (Table 2). The adjusted vaccine effectiveness among persons admitted to the hospital before Delta variant predominance (February 1–June 30) (84.1%; 95% CI = 74.1%–90.2%) was similar to vaccine effectiveness during Delta variant predominance (July 1–August 6) (89.3%; 95% CI = 80.1%–94.3%). The estimated vaccine effectiveness among persons aged ≥65 years (79.8%; 95% CI = 67.7%–87.4%) was lower than among persons aged 18–64 years (95.1%; 95% CI = 89.1%–97.8%), and no difference was found between persons who had completed the full vaccination series <90 days (86.1%; 95% CI = 76.5%–91.8%) versus ≥90 days (87.2%; 95% CI = 78.2%–92.5%) before their SARS-CoV-2 test date. Adjusted vaccine effectiveness estimates were also similar for Black (86.9%; 95% CI = 76.9%–92.6%) and White persons (88.1%; 95% CI = 77.4%–93.8%), as well as for Pfizer-BioNTech (83.4%; 95% CI = 74.0%–89.4%) and Moderna vaccines (91.6%; 95% CI = 83.5%–95.7%).

**TABLE 2:** Adjusted effectiveness* of full vaccination^†^ with mRNA COVID-19 vaccines against COVID-19–associated hospitalization among veterans, by characteristics of case-patients and controls — five Veterans Affairs Medical Centers,^§^ United States, February 1–August 6, 2021

Characteristic	n/N (%)		Adjusted vaccine effectiveness % (95% CI)
	Case-patients vaccinated/total	Controls vaccinated/total	
**Overall**	**54/388 (13.9)**	**378/787 (48.0)**	**86.8 (80.4–91.1)**
**Age group, yrs**			
18–64	10/199 (5.0)	93/275 (33.8)	95.1 (89.1–97.8)
≥65	44/189 (23.3)	285/512 (55.7)	79.8 (67.7–87.4)
**Race/Ethnicity^¶^**			
Black, non-Hispanic	24/195 (12.3)	169/379 (44.6)	86.9 (76.9–92.6)
White, non-Hispanic	21/141 (14.9)	171/334 (51.2)	88.1 (77.4–93.8)
**COVID-19 vaccine product among fully vaccinated**			
BNT162b2 (Pfizer-BioNTech)	43/388 (11.1)	242/787 (30.7)	83.4 (74.0–89.4)
mRNA-1273 (Moderna)	11/388 (2.8)	136/787 (17.3)	91.6 (83.5–95.7)
**Date of hospital admission**			
February 1–June 30	22/270 (8.1)	249/618 (40.3)	84.1 (74.1–90.2)
July 1–August 6	32/118 (27.1)	129/169 (76.3)	89.3 (80.1–94.3)
**No. of days since fully vaccinated**			
<90 days	19/388 (4.9)	215/787 (27.3)	86.1 (76.5–91.8)
≥90 days	35/388 (9.0)	163/787 (20.7)	87.2 (78.2–92.5)

**Abbreviation:** CI = confidence interval.

*All nonstratified models adjusted for study site, time (admission date), age, sex, and race/ethnicity. Stratified models exclude adjustment for stratification variable.

**^†^** Full vaccination was defined as receipt of both doses of an mRNA COVID-19 vaccine (Pfizer-BioNTech or Moderna) ≥14 days before the first SARS-CoV-2 test performed within 14 days before admission or during the first 72 hours of hospitalization.

^§^ Atlanta, Georgia; Bronx, New York; Houston, Texas; Los Angeles, California; and Palo Alto, California.

^¶^ Because of small numbers of veterans in other racial/ethnic groups, vaccine effectiveness was estimated only for non-Hispanic Black and non-Hispanic White persons.

## Discussion

Among U.S. veterans hospitalized at five VAMCs, mRNA vaccines were 86.8% effective in preventing COVID-19–associated hospitalizations and remained highly effective during a period of Delta variant predominance. The mRNA vaccines were effective against COVID-19–associated hospitalization among all age groups, although lower effectiveness (79.8%) was observed among veterans aged ≥65 years. These findings support current evidence that COVID-19 mRNA vaccines are highly effective in preventing COVID-19–associated hospitalization (*1*–*3*) and reinforce the importance of vaccination, including among veterans, who are at high risk for COVID-19 hospitalization because they are older and have a higher prevalence of underlying medical conditions compared with persons in the general U.S. population (*2*,*4*).

Consistent with national trends,^§§^ Delta became the predominant SARS-CoV-2 variant in this cohort in July 2021. Protection against COVID-19–associated hospitalization remained high despite the emergence of Delta as the predominant variant in the United States; protection was similar during periods before (February–June 2021; 84.1%) and during (July–August 2021; 89.3%) Delta variant predominance. Recent reports have shown that COVID-19 vaccine protection against SARS-CoV-2 infection is lower in areas with increasing Delta variant transmission (*7*,*8*); however, protection against severe disease outcomes, including hospitalization, remains high (*7*,*9*).

Although the observed vaccine effectiveness in this study is similar to that reported by other studies measuring protection against COVID-19–associated hospitalization, significantly lower vaccine effectiveness among older adults has not previously been observed (*1*,*2*,*9*). This might be a result of differences in the populations evaluated; periods of vaccine effectiveness assessment, including differences in vaccine coverage, variant circulation, and time since vaccination; and variability in unmeasured confounding. Decreased immunogenicity with increasing age has been reported after vaccination with COVID-19 mRNA vaccines (*10*). Because one fourth of adults included in this evaluation were aged ≥75 years, age-related differences in immunogenicity might have significantly contributed to lower estimated effectiveness in older persons. Additional evaluations of vaccine effectiveness across age groups, including the relationship between age and duration of protection, are warranted.

The findings in this report are subject to at least four limitations. First, although the five VAMCs included in this assessment were in diverse geographic locations, they are not representative of the entire veteran population or the general U.S. population. Second, despite the inclusion of 1,175 participants, the statistical power was insufficient to detect potential differences in vaccine effectiveness among all subgroups. Third, vaccine effectiveness estimates might be confounded by certain unmeasured behaviors, including mask use or time spent in congregate settings. Finally, the number of veterans in this sample who received the Janssen COVID-19 vaccine was too small to assess the effectiveness of this vaccine in preventing COVID-19–associated hospitalization.

These findings show that the COVID-19 mRNA vaccines remain highly effective for preventing COVID-19–associated hospitalization in this older, racially diverse population of predominantly male U.S. veterans, including during periods of widespread circulation of the SARS-CoV-2 Delta variant. However, vaccine effectiveness was lower among veterans aged ≥65 years than among those aged 18–64 years. Additional evaluations, particularly among older adults with high prevalences of underlying conditions, are important to assess vaccine effectiveness in these populations. COVID-19 vaccination of all eligible persons is essential to prevent COVID-19–associated hospitalizations.

**Summary**

**What is already known about this topic?**

mRNA COVID-19 vaccines are effective in preventing severe COVID-19 outcomes, including hospitalization.

**What is added by this report?**

During February 1–August 6, 2021, vaccine effectiveness among U.S. veterans hospitalized at five Veterans Affairs Medical Centers was 87%. mRNA COVID-19 vaccines remain highly effective, including during periods of widespread circulation of the SARS-CoV-2 B.1.617.2 (Delta) variant. Vaccine effectiveness in preventing COVID-19–related hospitalization was 80% among adults aged ≥65 years compared with 95% among adults aged 18–64 years.

**What are the implications for public health practice?**

To protect against COVID-19–related hospitalization, all eligible persons should receive COVID-19 vaccination. Additional studies are needed to understand differences in COVID-19 vaccine effectiveness across age groups.

## Footnotes

* These authors contributed equally to this report.

† The test-negative study design included controls with the same clinical syndrome as case-patients to reduce bias from differences in health care–seeking behavior as well as access to testing and care.

§
https://www.fda.gov/media/134922/download

¶
https://www.who.int/publications/i/item/WHO-2019-nCoV-vaccine_effectiveness-measurement-2021.1

** 45 C.F.R. part 46.102(l)(2), 21 C.F.R. part 56; 42 U.S.C. Sect. 241(d); 5 U.S.C. Sect. 552a; 44 U.S.C. Sect. 3501 et seq.

†† Among case-patients with COVID-19–like illness and any COVID-19 vaccination status.

§§
https://covid.cdc.gov/covid-data-tracker/#variant-proportions

## References

[1] Tenforde MW, Patel MM, Ginde AA, Effectiveness of SARS-CoV-2 mRNA vaccines for preventing Covid-19 hospitalizations in the United States. Clin Infect Dis 2021. Epub Aug 6, 2021. 34358310

[2] Young-Xu Y, Korves C, Roberts J, Coverage and effectiveness of mRNA COVID-19 vaccines among veterans. medRxiv [Preprint posted online July 14, 2021]. https://www.medrxiv.org/content/10.1101/2021.06.14.21258906v3 10.1001/jamanetworkopen.2021.28391 PMC8495523 34613401

[3] Dagan N, Barda N, Kepten E, BNT162b2 mRNA Covid-19 vaccine in a nationwide mass vaccination setting. N Engl J Med 2021;384:1412–23. 10.1056/NEJMoa2101765 33626250 PMC7944975

[4] Cardemil CV, Dahl R, Prill MM, COVID-19–related hospitalization rates and severe outcomes among veterans from 5 Veterans Affairs Medical Centers: hospital-based surveillance study. JMIR Public Health Surveill 2021;7:e24502. 10.2196/24502 33338028 PMC7836907

[5] King JT Jr, Yoon JS, Rentsch CT, Development and validation of a 30-day mortality index based on pre-existing medical administrative data from 13,323 COVID-19 patients: The Veterans Health Administration COVID-19 (VACO) Index. PLoS One 2020;15:e0241825. 10.1371/journal.pone.0241825 33175863 PMC7657526

[6] Paden CR, Tao Y, Queen K, Rapid, sensitive, full-genome sequencing of severe acute respiratory syndrome coronavirus 2. Emerg Infect Dis 2020;26:2401–5. 10.3201/eid2610.201800 32610037 PMC7510745

[7] Rosenberg ES, Holtgrave DR, Dorabawila V, New COVID-19 cases and hospitalizations among adults, by vaccination status—New York, May 3–July 25, 2021. MMWR Morb Mortal Wkly Rep 2021;70:1150–5. 10.15585/mmwr.mm7034e1 34437517 PMC8389393

[8] Nanduri S, Pilishvili T, Derado G, Effectiveness of Pfizer-BioNTech and Moderna vaccines in preventing SARS-CoV-2 infection among nursing home residents before and during widespread circulation of the SARS-CoV-2 B.1.617.2 (Delta) variant—National Healthcare Safety Network, March 1–August 1, 2021. MMWR Morb Mortal Wkly Rep 2021;70:1163–6. 10.15585/mmwr.mm7034e3 34437519 PMC8389386

[9] Tenforde MW, Self WH, Naioti EA, ; IVY Network Investigators. Sustained effectiveness of Pfizer-BioNTech and Moderna vaccines against COVID-19 associated hospitalizations among adults—United States, March–July 2021. MMWR Morb Mortal Wkly Rep 2021;70:1156–62. 10.15585/mmwr.mm7034e2 34437524 PMC8389395

[10] Collier DA, Ferreira IATM, Kotagiri P, ; CITIID-NIHR BioResource COVID-19 Collaboration. Age-related immune response heterogeneity to SARS-CoV-2 vaccine BNT162b2. Nature 2021;596:417–22. 10.1038/s41586-021-03739-1 34192737 PMC8373615

